# The effect of replacing 1 week of content teaching with an intensive simulation-based learning activity on physiotherapy student clinical placement performance

**DOI:** 10.1186/s41077-019-0095-8

**Published:** 2019-12-20

**Authors:** Neil Tuttle, Sean A. Horan

**Affiliations:** 1 School of Allied Health Sciences, Gold Coast campus, Griffith University, Gold Coast, QLD 4222 Australia; 2 Menzies Health Institute Queensland, Gold Coast, Australia

**Keywords:** Clinical placement, Physiotherapy, Simulated learning, Curriculum design

## Abstract

**Background:**

Simulation-based learning (SBL) activities are increasingly used to replace or supplement clinical placements for physiotherapy students. There is limited literature evaluating SBL activities that replace on-campus teaching, and to our knowledge, no studies evaluate the role of SBL in counteracting the negative impact of delay between content teaching and clinical placements. The aims of this study were to (i) determine the effect on clinical placement performance of replacing 1 week of content teaching with a SBL activity and (ii) determine if a delay between content teaching and clinical placement impacted clinical placement performance.

**Methods:**

This study is a retrospective cohort study. Participants included students in the first two clinical placements of a graduate-entry, masters-level program. Six hundred twenty-nine student placements were analysed—285 clinical placements where students undertook a 20-h SBL activity immediately prior to clinical placement were compared with 344 placements where students received traditional content. Of the placements where students received the SBL, 147 occurred immediately following content teaching and 138 had a delay of at least 5 weeks. Performance on clinical placement was assessed using the Assessment of Physiotherapy Practice (APP).

**Results:**

There was a significant main effect of SBL with higher APP marks for the experimental group (3.12/4, SD = 0.25 vs 3.01/4, SD = 0.22), and post hoc analysis indicated marks were significantly higher for all seven areas of assessment. Students whose placements immediately followed content teaching performed better on mid-placement APP marks in two areas of assessment (analysis and planning, and intervention) compared to students for whom there was a delay. There were no statistically significant differences in relation to delay for end of placement APP marks.

**Conclusion:**

Replacing 1 week of classroom teaching with a targeted, SBL activity immediately before placement significantly improved student performance on that clinical placement. A negative impact of delay was found on mid-placement, but not the end of placement APPs. Findings of improved performance when replacing a week of content teaching with a targeted SBL activity, and poorer performance on mid-placement marks with a delay between content teaching and clinical placement, may have implications for curriculum design.

## Background

This paper describes the evaluation of a simulation- based learning (SBL) activity that was developed for physiotherapy students with two purposes: first, to act as a transition between content teaching and clinical placement, and second, to act as a refresher for students who have a delay between content teaching and their related clinical placements.

### The role of SBL in physiotherapy education

The use of SBL activities in health professional education has historically focused on teamwork and exposure to high-risk situations. These activities have often taken the form of complex, interdisciplinary, high-fidelity scenarios [1]. More recently, however, SBL activities have increasingly been integrated into more traditional classroom activities.

A strong driver of SBL in physiotherapy education is the increasing demands on clinical placement capacity [2]. Consequently, SBLs have been used to replace clinical placement time [3]. A less commonly reported and potentially more viable approach is to have SBL activities replace on-campus teaching, with the intention of better preparing students for clinical placement [3].

### Evaluation of SBL activities

Change in student confidence is the most common way of evaluating SBL activities in physiotherapy education [4] and would be considered by Kirkpatrick as the second of four levels of evaluation of learning [5]. Change in confidence however may be a poor indicator of educational effectiveness because (i) student confidence can decrease as they gain more understanding [6], (ii) more confident students typically overestimate their level of competence [7] potentially making them more likely to engage in ‘risky’ clinical behaviours [8], and (iii) poorer placement outcomes occur when there is a mismatch between physiotherapy student self-assessment and educator assessment [9].

A more meaningful method of evaluating the effectiveness of SBL activities that has been used in several studies within physiotherapy is comparing student marks on clinical placement [4]. The Assessment of Physiotherapy Practice (APP), which is a reliable [10] and valid [11] instrument that is used to evaluate all physiotherapy clinical placements in Australia, is perhaps the most commonly reported assessment tool. Such evaluation of performance on clinical placement would generally be considered as part of the third level of Kirkpatrick’s hierarchy behaviour.

### Effect of SBL on clinical placement performance

SBL has been shown to be able to replace up to 25% of clinical placement time without adversely impacting student outcomes [12]. In another related study, although the authors rightly suggest caution in interpretation of their findings, replacing an introductory placement with a simulated placement appeared to produce superior results [13].

Not all simulations within physiotherapy education, however, are used to replace clinical placement time. As of 2015 in Australia, the same number of universities were planning on using simulation within their academic programs as were planning on using SBL to replace clinical placement hours [2]. The impact on clinical placement of replacing on-campus content teaching with an SBL activity has been evaluated in at least one previous study [14]. In this study, student performance on clinical placement improved following a brief SBL activity focused on improving student skills in providing and responding to feedback. Consequently, we incorporated a similar feedback module into the SBL activity that is the subject of this manuscript.

### Timing of SBL and clinical placements

For many physiotherapy programs, there is a delay between content learning and application of that learning in relevant clinical placements. In disciplines other than physiotherapy, skills have been shown to deteriorate after periods greater than 1 month [15, 16]. Within physiotherapy, student clinical placement performance was found to deteriorate when there was a delay of five or more weeks between content teaching and clinical placements [17]. It is unknown whether the inclusion of an SBL module immediately before each student’s placement would mitigate the effect of delay between content teaching and the related clinical placement.

### Aims

The primary aim of this study was to determine the effect on clinical placement performance of replacing 1 week of traditional on-campus content teaching with 1 week of SBL. A secondary aim was to determine if a week of SBL immediately before clinical placement counteracted the negative effect of delay on clinical placement performance.

## Methods

**Fig. 1:**
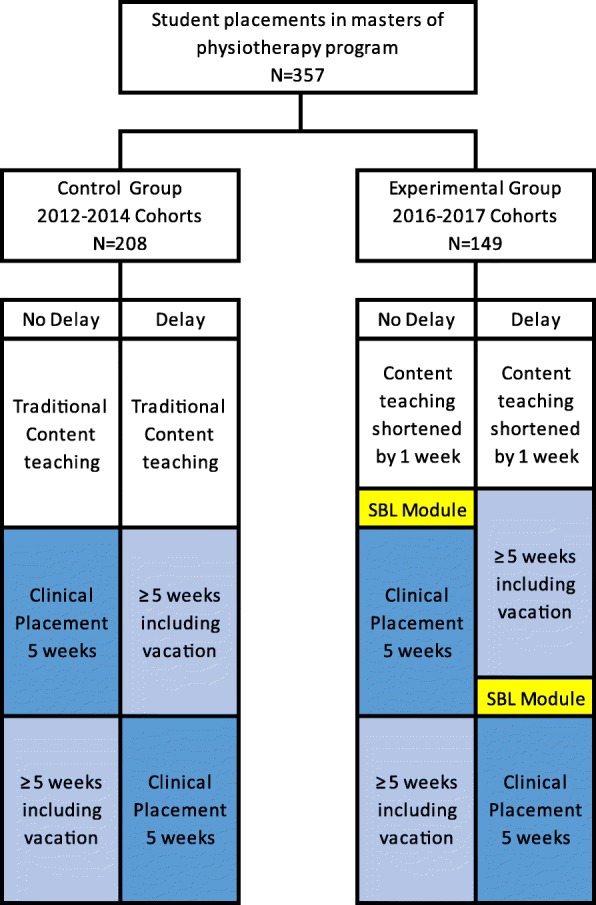
Flow chart of participants

A retrospective cohort study was used to evaluate the physiotherapy student performance on clinical placement. The performance of students who received traditional content teaching was compared with others where the final week of content teaching was replaced with a targeted SBL activity timed to occur immediately prior to each student’s clinical placement. To address the second aim, for students who undertook the SBL immediately prior to their clinical placement, the performance of those whose clinical placement occurred immediately after their on-campus teaching was compared with students who had a delay between their on-campus teaching and their clinical placement. Figure 1 shows the flow of students in the various groups.

### Participants

Students in the first year of a 2-year graduate entry master of physiotherapy program participated in the study. The program utilises an integrated clinical placement model, where clinical placement blocks follow the relevant on-campus teaching content. In the program, students undertake a total of five, 5-week clinical placements. The first two of these blocks are now preceded by an SBL module and are the subject of this study. Students who were repeating the preceding course or repeating clinical placements were excluded from the analysis (total student placements *n* = 629).

### Procedures and evaluation

To examine the effect of the SBL module on student performance, we retrospectively compared the end of placement marks for students who undertook the SBL prior to their placements (experimental group: cohorts from 2016 to 2017, *n* = 285) with a group of students who did not receive the SBL module (control group: cohorts from 2012 to 2014, *n* = 344). The teaching content was comparable between the cohorts except for the replacement of 1 week of on-campus teaching with the SBL module. Mid-placement marks were not considered in the analysis because they were mandatory for the experimental group, but optional for the control group. The cohort between those being analysed (2015) included a partial SBL experience so was not included in the study.

To address the second aim, we considered only the experimental group and compared the mid-placement and end of placement marks for students who undertook their SBL and placement immediately after on-campus content (no delay, *n* = 138) with those who had a delay of five or more weeks between content teaching and clinical placement (delay, *n* = 147).

The performance of students on clinical placement was evaluated with the Assessment of Physiotherapy Practice (APP). The APP includes twenty items each marked on a five-level (0–4) scale. The items are categorised into seven sections: (i) professional behaviour, (ii) communication, (iii) assessment, (iv) analysis and planning, (v) intervention, (vi) evidence-based practice, and (vii) risk management. The authors considered the experimental and control groups to be sufficiently similar to allow a direct comparison even though different cohorts of students were compared because (i) clinical placements occurred within a relatively stable pool of hospitals and outpatient clinics, (ii) average APP marks across all Australian universities had not systematically altered in that time, and (iii) APP marks for the three subsequent placements of Griffith University students did not change significantly across the time period included in this study.

### Simulation-based learning activity

The SBL activity whose impact is being investigated in this manuscript is described in detail elsewhere [18], so only a brief description will be presented here. The module was developed as an intensive activity across 1 week, with the purpose of preparing students for clinical placement. It was not an extension of teaching time, rather an activity which replaced traditional content teaching. The module occurred in the week immediately prior to each of the first two clinical placements for each student. The first clinical placement was in an orthopaedic inpatient setting in the first semester of the program following teaching of relevant on-campus content. The second clinical placement was in a musculoskeletal outpatient setting at the end of the second semester (i.e. end of year one), again after completing relevant on-campus content. For a given student, each placement can occur either immediately after the relevant on-campus content or with a delay of at least 5 weeks, during which time students have vacation time and in some cases non-clinical on-campus content.

The settings for each of the SBL modules replicated as closely as possible the setting the students would be in the following week including relevant patient scenarios, appropriate beds and equipment. The SBL activities were located at the Griffith University, Gold Coast campus. Pre-briefing, debriefing, and student independent activities related to the module were conducted away from the simulation space. Students worked in a consistent group of four students for the week but worked with different facilitators on different days. The facilitators were university teaching staff or experienced clinical educators. Where possible, all facilitators undertook specific online and workshop training in simulation methodologies [19].

Students participated for approximately 20 h spread over the 5 days. On each day, students saw two or three patients including new and follow-up consultations. Scenarios were informed by a stakeholder audit [20], with each day having a focus of either an aspect of patient care (e.g. inter-professional communication or risk minimisation) or a component of the patient interaction (e.g. taking a history or physical assessment) [18]. Some background or follow-up material was presented in an interactive online format to enable the live simulation to be more specifically targeted [21]. Actors experienced in simulation were recruited to match the demographic profile for each scenario and were rehearsed prior to participation. On each day, 1–4 actors would be portraying each patient, 4–8 clinical educators would be facilitating, and 32–48 students would be working in groups of four. The scenarios were intended to be ‘on the edge’ of student expertise [22] by simulating the type of clinical placement setting students would undertake the following week. An important difference between the module and simulating clinical practice is that, unlike in clinical practice, students were expected to regularly consult with their clinical educator (portrayed by their simulation facilitator) during the patient consultations. A variety of debriefing strategies were employed by the facilitators [23] and the simulated patients (SPs) provided feedback on student communication skills. In many of the sessions, there was the opportunity for either the same or a different student to redo all or part of the live interaction with the SP. In spite of there being no summative assessment during the week, students were highly motivated by knowing they would be in a similar real-life situation the following week.

### Data analysis

The percentage of students who failed their clinical placement was calculated for each group. Analysis of placement marks was undertaken using two separate ANOVAs (GLM). To examine the effect of the module, we compared the end of placement APP marks for each of the seven sections between the experimental and control groups with delay, order of placement (first or second clinical placement), and area of practice as additional factors. To examine the effect of delay, we compared mid-placement and end of placement APP marks for placements where there was a delay (delay) and not a delay (no delay) with placement (whether it was the students’ first or second placement) as a factor. Analysis was undertaken in SPSS® (IBM Corp. Released 2013. Version 22.0. Armonk, NY: IBM Corp). Significance levels were set at *p* < 0.05.

## Results

### Effect of simulation

**Fig. 2:**
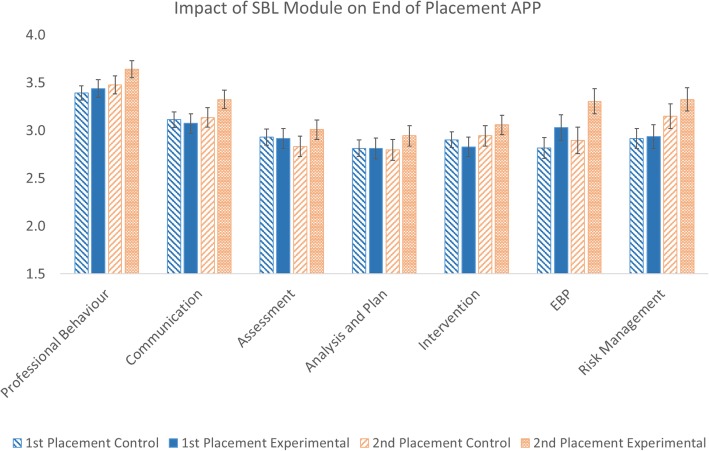
Experimental and control group APP marks

Clinical placement failure rates were not significantly different between the experimental (3.5%) and control (3.7%) groups. There was a significant main effect of the SBL module (*p* < 0.001) with APP marks being higher than the control group (3.12/4, SD = 0.25 vs 3.01/4, SD = 0.22). Post hoc analysis indicated that marks were significantly higher for all seven sections of the APP for the experimental group (Fig. 2).

### Effect of delay in the presence of simulation

**Fig. 3:**
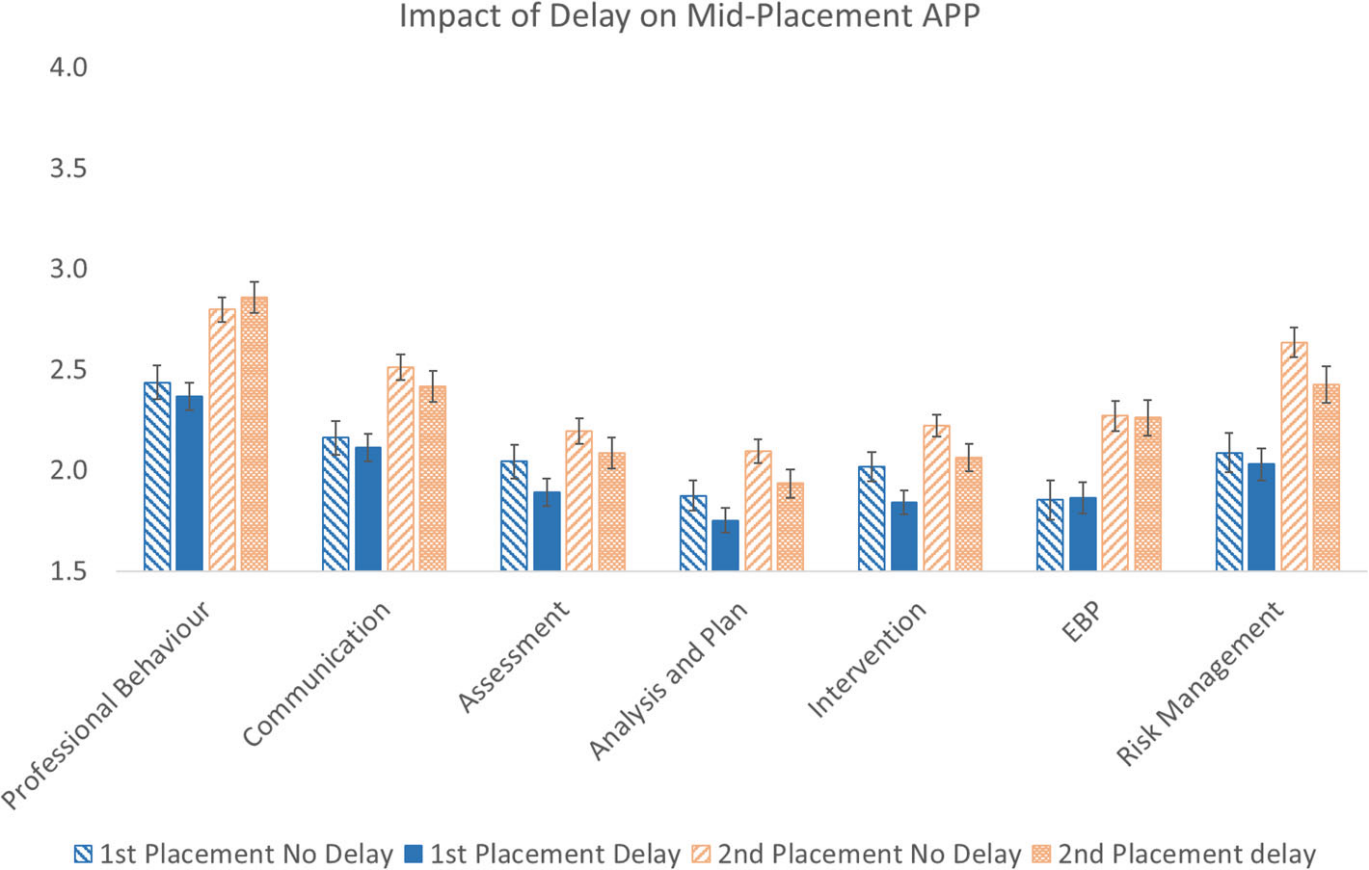
Delay/no delay APP marks at mid-placement for the first and second placements

For mid-placement marks, a main effect of delay approached significance (*p* = 0.051) with post hoc examination revealing a significant effect on mid-placement marks for two assessment areas: analysis and planning (*p* = 0.034) and intervention (*p* = 0.009) (Fig. 3).

**Fig. 4:**
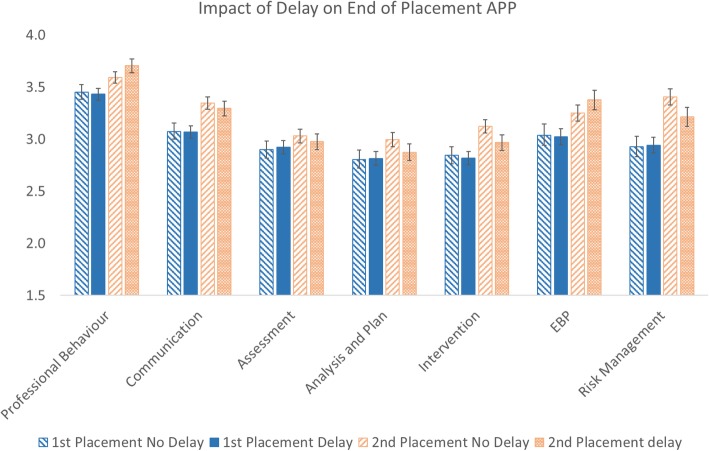
Delay/no delay marks at end-of-placement for the first and second placements

For the end of placement APP marks, no significant difference was found for delay either as a main effect (*p =* 0.476) or for any of the seven sections of the APP (Fig. 4).

## Discussion

The findings from this study indicate that students who received the targeted SBL module immediately before their clinical placements performed significantly better at their corresponding end of placement assessment than previous cohorts. Students whose placement occurred immediately after their content teaching performed better on the mid-placement APP for two out of the seven sections. There were no differences in end of placement APP marks for students without or with delay.

### Effectiveness of SBL activities

To our knowledge, this study is the first investigation within physiotherapy education to evaluate an intensive SBL activity lasting more than 1 day that replaced on- campus content rather than replacing clinical placement time. Previous studies have reported that replacing clinical placement time with SBL was not inferior to traditional placements [4]. Previous studies of short SBL activities that occurred outside of clinical placement time demonstrated either no effect [24] or a positive effect [14] on clinical placement performance. More recently, it has been suggested that the replacement of some clinical placement time with simulation may indeed produce superior results to traditional placements [13] and this study confirms that superior results are possible.

One point of difference between the SBL module used in this study and some others was that in this study, the SBL was structured specifically to prepare students for clinical placement as distinct from preparing them for clinical practice. As such, the SBL included the interactions with clinical educators as would occur on clinical placement which would be within students’ zone of proximal development [25]. Many of the interactions between students and educators are specific to clinical placements and are considered a critical skill set for optimal learning in the clinical placement setting. In the early stages of clinical placements, students are not expected to operate independently [26]. Rather, students have interactions with their clinical educator that include providing verbal and/or written summaries of impressions, reasoning, and plans; responding to questions and feedback; and revising parts of the interactions with their patients. These learning conversations are an important contributor to student learning while on placements. Students frequently respond ineffectively or even counterproductively to such conversations [14]. The SBL module in this study directly provided a conceptual framework for such conversations and enabled students to develop skills to increase the effectiveness of these conversations and thereby their learning while on placement. These deep and authentic learning experiences tend to intrinsically motivate students and were part of the rationale for replacing classroom content with an SBL activity in this study. For many of the previous studies, it is unclear whether the SBL activity targeted improvement in clinical placement or independent practice. The authors believe that there are significant implications of what skills are targeted and therefore the setting that is simulated.

One of the major drivers for SBL in physiotherapy education has been to counteract increasing demands on limited clinical placement capacity. It may therefore seem counterintuitive to put extensive time and resources into SBL activities that do not directly replace clinical placement time. There are however ways that SBL activities that do not replace clinical placement hours can be effective in decreasing the strain on clinical placement capacity. Hosting student placements can be made more attractive to potential clinical placement facilities. If students perform better on placement, particularly by increasing their independence earlier, then students may be more attractive to placement facilities either because of reduced load on the clinical educators or through increased occasions of service. Neither of these variables were evaluated in this study.

#### Delay

Consistent with previous findings, some areas of students’ mid-placement performance were poorer if there was a delay between their relevant academic content and their clinical placement. Although it was not assessed in this study, mid-placement marks may provide an indication of how quickly students become more independent and therefore impact on how advantageous students are to the host facility. The areas of assessment that were lower with delay for this study, however, were different from previous findings. Our previous study found three areas (professional behaviour, evidence-based practice, and risk management) were lower following delay, while this study found two areas (analysis and planning and intervention). Judd and colleagues recently found that two areas, when assessed in simulation, predicted student performance on subsequent clinical placements [27]. One of these areas (risk assessment) had significantly deteriorated following delay in our previous study, and another area (analysis and planning) had a significant deterioration in this study. While further investigation is required, it is possible that these two areas may be predictive of performance more generally. In summary, there appears to be a negative effect of delay that is apparent in the first half of clinical placements, but the areas of performance where this occurs are not consistent.

#### Limitations

This study evaluated the effect of a specific and targeted SBL activity on clinical placement performance. The results are therefore not necessarily generalisable to other SBL modules. An important limitation of this study is that the comparisons to determine the effectiveness of the SBL module were between cohorts from different years. Although the only known difference between the cohorts was the presence of the SBL activity, it cannot be certain that other differences were not present between the groups themselves or in other aspects of their education. To evaluate whether there were other performance differences between groups, the grades in the on-campus courses were compared between the groups, but no differences were found. Further, to determine if there may have been “marks inflation” across clinical placements, APP marks across all universities in Australia were compared and no significant differences were found between the years that were considered in this study. Another limitation is inherent in the responsiveness of the APP itself. It may be that there is a ceiling effect particularly for the end of placement marks which would make the end of placement APP less sensitive to, for example, effects of delay.

## Conclusions

A 1-week targeted SBL activity immediately prior to the first two clinical placements for physiotherapy students in an entry-level program appeared to result in global improvements in performance on clinical placement. Although it had been anticipated that the SBL activity might counteract the negative effect on mid-placement performance of a delay between content teaching clinical placement, this was not the case. SBL activities vary considerably both in their content and execution. The authors advise caution in generalising the findings of improvement following inclusion of SBL activities found in this study to other SBL activities. The authors suggest that the modest negative effect of delay between content teaching and clinical placements found in this and previous studies has implications for curriculum design.

## Abbreviations

APP: Assessment of Physiotherapy Practice
SBL: Simulation-based learning
SP(s): Simulated patient(s)

## References

[1] Rosen KR (2008). The history of medical simulation. J Crit Care.

[2] Wright A, Moss P, Watson K, Rue S, Jull G, Mandrusiak A, Reubenson A, Connaughton J, Redmond C, MacIntosh S (2015). A profession-wide collaboration to embed role-play simulation into Australian entry-level physiotherapy clinical training. Physiotherapy.

[3] Pritchard SA, Blackstock FC, Nestel D, Keating JL (2016). Simulated patients in physical therapy education: a systematic review and meta-analysis. Phys Ther.

[4] Mori B, Carnahan H, Herold J (2015). Use of simulation learning experiences in physical therapy entry-to-practice curricula: a systematic review. Physiother Can.

[5] Praslova L (2010). Adaptation of Kirkpatrick’s four level model of training criteria to assessment of learning outcomes and program evaluation in higher education. Educ Assess Eval Account.

[6] Starrels JL, Fox AD, Kunins HV, Cunningham CO (2012). They don’t know what they don’t know: internal medicine residents’ knowledge and confidence in urine drug test interpretation for patients with chronic pain. J Gen Intern Med.

[7] Dunning D, Heath C, Suls JM (2004). Flawed self-assessment: implications for health, education, and the workplace. Psychol Sci Public Interest.

[8] Davis DA, Mazmanian PE, Fordis M, Van Harrison R, Thorpe KE, Perrier L (2006). Accuracy of physician self-assessment compared with observed measures of competence: a systematic review. JAMA.

[9] Lo K, Osadnik CR, Leonard M, Maloney SR (2016). Student–clinician agreement in clinical competence as a predictor of clinical placement performance in Australian undergraduate physiotherapy students. Physiother Theory Pract.

[10] Dalton M, Davidson M, Keating JL (2012). The assessment of physiotherapy practice (APP) is a reliable measure of professional competence of physiotherapy students: a reliability study. J Physiother.

[11] Dalton M, Davidson M, Keating J (2011). The assessment of physiotherapy practice (APP) is a valid measure of professional competence of physiotherapy students: a crosssectional study with Rasch analysis. J Physiother.

[12] Watson K, Wright A, Morris N, McMeeken J, Rivett D, Blackstock F, Jones A, Haines T, O’Connor V, Watson G (2012). Can simulation replace part of clinical time? Two parallel randomised controlled trials. Med Educ.

[13] Wright A, Moss P, Dennis DM, Harrold M, Levy S, Furness AL, Reubenson A (2018). The influence of a full-time, immersive simulation-based clinical placement on physiotherapy student confidence during the transition to clinical practice. Adv Simul (Lond).

[14] Tuttle N, Bialocerkowski A (2017). Developing student skills to actively engage in feedback conversations: a pilot study. Internet J Allied Health Sci Pract.

[15] Ginzburg S, Dar-El E (2000). Skill retention and relearning–a proposed cyclical model. J Work Learn.

[16] James B, Beattie M, Shepherd A, Armstrong L, Wilkinson J (2016). Time, fear and transformation: student nurses’ experiences of doing a practicum (quality improvement project) in practice. Nurse Educ Pract.

[17] Horan S, Tuttle N. Does delay between teaching content and related clinical placements affect performance in physiotherapy students? In: ANZAHPE Conference 2018. Hobart Tasmania; ANZAHPE; 2018.

[18] Tuttle N, Laakso EL. Simulated learning environments to prepare for clinical placements: Transition to placement (T2P). In: Sing I, Raghuvanshi, K, editors. Emerging Technologies and Work- Integrated Learning Experiences in Allied Health Education. Hershey, PA.: IGI Global; 2018. p. 180–207.

[19] The National Health Education and Training - Simulation Program. https://www.monash.edu/medicine/nhet-sim. Accessed 16 May 2019.

[20] Tuttle N, Edwards S: Using stakeholder input to inform scenario content of simulated learning environments: an example case. Adv Simul (Lond) Under review 10.1186/s41077-019-0102-0 PMC6923834 31890316

[21] Tuttle N. The Use of an Online Adaptive Learning Platform as an Adjunct to Live Simulated Clinical Encounters. In: Sing I, Raghuvanshi, K, editors. Emerging Technologies and Work- Integrated Learning Experiences in Allied Health Education. Hershey, PA; 2018. p. 93–105.

[22] Rudolph JW, Raemer DB, Simon R (2014). Establishing a safe container for learning in simulation: the role of the presimulation briefing. Simul Healthc.

[23] Eppich W, Cheng A (2015). Promoting excellence and reflective learning in simulation (PEARLS): development and rationale for a blended approach to health care simulation debriefing. Simul Healthc.

[24] Jones A, Sheppard L (2011). Use of a human patient simulator to improve physiotherapy cardiorespiratory clinical skills in undergraduate physiotherapy students: a randomised controlled trial. Internet J Allied Health Sci Pract.

[25] Sanders D, Welk DS (2005). Strategies to scaffold student learning: applying Vygotsky’s zone of proximal development. Nurse Educ.

[26] Clouder L, Adefila A (2017). Empowerment of physiotherapy students on placement: the interplay between autonomy, risk, and responsibility. Physiother Theory Pract.

[27] Judd B, Fethney J, Alison J, Waters D, Gordon C (2018). Performance in simulation is associated with clinical practice performance in physical therapist students. J Phys Ther Educ.

